# Effect of Pulsed Field Timing Sequence and Electrode Cooling on Ablation Safety and Efficacy Surrogates: A Preclinical Study

**DOI:** 10.1161/CIRCEP.126.015042

**Published:** 2026-09-23

**Authors:** Luigi Di Biase, Jonathan Hsu, Rahul Bhardwaj, David Newton, William Maddox, Jorge E. Romero, Tara Gomez, Eric Byun, Qi Chen, Christopher Beeckler, Fengwei Zou, William H. Sauer

**Affiliations:** 1 Department of Cardiology, Montefiore-Einstein Center for Heart and Vascular Care, Montefiore Medical Center, Bronx, NY (L.D.B., F.Z.).; 2 Cardiac Electrophysiology Section, Division of Cardiology, Department of Medicine, University of California, San Diego (J.H.).; 3 Departments of Cardiology and Electrophysiology, Loma Linda University Medical Center, Loma Linda University International Heart Institute, CA (R.B.).; 4 Clinical Cardiac Electrophysiology Department, Memorial Health University Medical Center, Savannah, GA (D.N.).; 5 University of Alabama at Birmingham (W.M.).; 6 Heart and Vascular Institute, Upstate Medical University, Syracuse, NY (J.E.R.).; 7 Johnson & Johnson MedTech, Irvine, CA (T.G., E.B., Q.C., C.B.).; 8 Cardiac Arrhythmia Service, Brigham and Women’s Hospital, Boston, MA (W.H.S.).

**Keywords:** animals, atrial fibrillation, catheter ablation, hemolysis, microbubbles

## Abstract

**BACKGROUND:**

The variable loop circular catheter has been shown to be a safe, effective pulsed field ablation catheter for atrial fibrillation treatment. After the commercial launch of the initial waveform (Seq1), a new waveform (Seq2_30 mL/min_) was developed that reduces electrode surface temperature and ablation delivery time (20–3.8 seconds). This study compares the safety and efficacy of Seq1 versus Seq2 across 2 irrigation rates and 2 dosing conditions.

**METHODS:**

Twenty-four Yorkshire pigs were treated with pulsed field ablation using the variable loop circular catheter. Subjects were randomized into 3 pulse recipe/irrigation arms (Seq1_4 mL/min_, Seq1_30 mL/min_, and Seq 2_30__mL/min_). Each group was split into a 1× nominal and a 2×-dose group, resulting in 6 total study groups. Animals were monitored for 28±2 days postablation for follow-up remapping procedures before euthanization. Gross necropsy and histopathology were performed on day 28±2.

**RESULTS:**

In all groups, 100% acute and chronic pulmonary vein isolation effectiveness was observed, as indicated by transmural, circumferential, pulmonary vein lesions with no evidence of collateral injury. Transmurality in the left atrial appendage, LA roof, and posterior wall was 75% to 100% across all groups and absent at the mitral isthmus. No catheter thrombus/char was observed at Seq2_30 mL/min_ or Seq1_30 mL/min_. With Seq1_4 mL/min_, thrombus/char occurred in 25% (1×) and 100% (2×). Hemolysis and myocardial injury marker changes were similar between all groups, with a notable increase in the 2× groups. On echocardiography, Seq2_30 mL/min_ microbubbles were rated as few or none, while Seq1_4 mL/min_ and Seq1_30 mL/min_ were mostly rated moderate or shower.

**CONCLUSIONS:**

Consistently durable pulmonary vein isolation was observed across all groups, including Seq1_4 mL/min_, Seq2_30 mL/min_, and Seq1_30 mL/min_ across both dosing conditions. Seq2_30 mL/min_ was associated with a reduced ablation delivery time (3.8 seconds), zero catheter thrombus formation, and few microbubbles. Higher irrigation flow in Seq1_30 mL/min_ also mitigated catheter thrombus formation but did not reduce microbubble generation.

**WHAT IS KNOWN?**

- First- and second-generation pulsed field ablation devices have emerged to further enhance the safety and efficacy of pulmonary vein isolation.
- The variable loop circular catheter initial pulse timing recipe (sequence 1) for pulmonary vein isolation has been shown to be safe and efficacious in both preclinical models and prospective clinical studies.

**WHAT THE STUDY ADDS**

- A new variable loop circular catheter waveform, called sequence 2, has been developed that reduces the thermal profile while also allowing a reduced ablation delivery time from 20.6 seconds (sequence 1) to 3.8 seconds.
- This study provides preclinical evidence that both sequence 2 and sequence 1 are effective and safe at the 1× and 2× therapeutic recommendations.
- Higher irrigation flow in sequence 1 also mitigated catheter thrombus formation.

Pulmonary vein isolation (PVI) remains the cornerstone catheter-based strategy for managing atrial fibrillation.^1^ Recent advancements in ablation technology have focused on enhancing both the effectiveness and safety of PVI, notably through first- and second-generation pulsed field ablation (PFA) devices.^2^

A first-generation variable loop circular catheter (VLCC) has been used globally for atrial fibrillation, utilizing a pulse sequence known herein as sequence 1; its first commercial iteration used continuous irrigation of 4 mL/min (Seq1_4 mL/min_).^3^ After commercial release, the irrigation rate for Seq1 increased to 30 mL/min (Seq1_30 mL/min_) to improve its thermal profile. Subsequent testing demonstrated that this higher rate delivered both clinical (reduced silent cerebral events) and performance (lower electrode heating) benefits.^4–9^

Building on that improvement, a second-generation VLCC pulse waveform was developed to further optimize the thermal profile while maintaining the lesion efficacy of Seq1_30 mL/min_. With electrodes cooling more effectively at the higher irrigation rate, sequence 2 (Seq2_30 mL/min_) shortens the time between pulses and overall energy delivery, reducing ablation duration from 20.6 seconds (Seq1) to 3.8 seconds.

Accordingly, this Good Laboratory Practice preclinical study aims to assess the safety and efficacy of the novel VLCC Seq2_30 mL/min_ waveform and existing commercial pulse sequences (Seq1_4 mL/min_ and Seq1_30 mL/min_) in a simulated clinical workflow.

## Methods

All relevant data are available in the main text or the Supplemental Material. All raw source files are available upon reasonable request from the corresponding author. This study was conducted in compliance with Good Laboratory Practice guidelines. All animal work adhered to the US Department of Agriculture Animal Welfare Act. The study protocol received approval from the institutional animal care and use committee and followed the American Heart Association guidelines for research animal use. The data that support the findings of this study are available from the corresponding author upon reasonable request.

### Seq2_30 mL/min_ Parameters

Compared with Seq1, Seq2 uses shorter pulse packets while keeping the same voltage (1800 V) and delivering biphasic, bipolar energy between alternate and adjacent electrodes. The interablation delay is shortened from 10 to 1 second and the ablation duration from ≈20 to 3.8 seconds. Lastly, Seq2 utilizes continuous irrigation at 30 mL/min during ablation and reduces to 4 mL/min during idle. Specific comparisons between waveforms are further defined in Figure 1.

**Figure 1.**
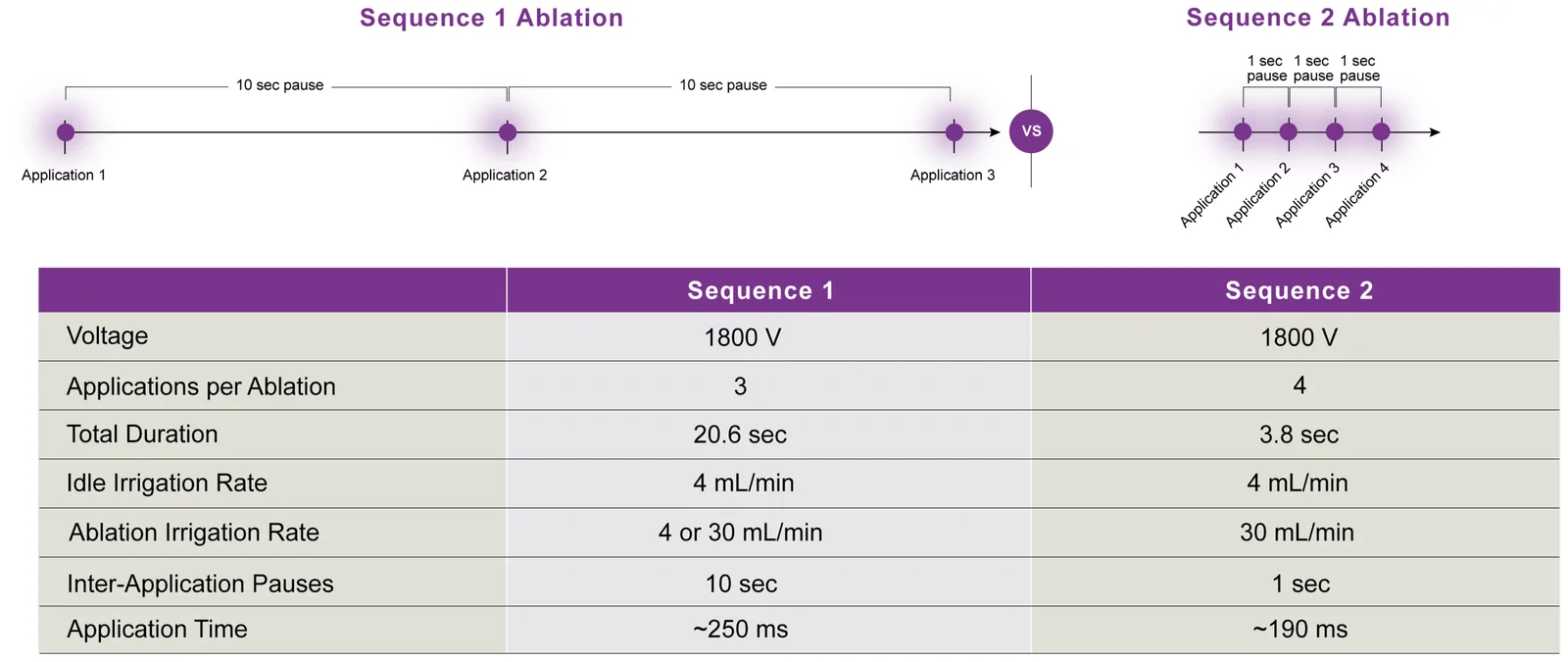
**Parameters of sequence 1 and sequence 2.** Detailed specifications for the 2 waveforms. Image is courtesy of Biosense Webster, Inc, part of Johnson & Johnson MedTech. © All rights reserved.

### Study Design and Ablation Strategy

Twenty-four (24) Yorkshire pigs were subject to PFA procedures using the TRUPULSE Generator (Biosense Webster, Inc, part of Johnson and Johnson MedTech, Irvine, CA) and VLCC (Varipulse, Biosense Webster, Inc, part of Johnson and Johnson MedTech, Irvine, CA).

Animals were first randomized into 3 primary test arms: (Seq1_4 mL/min_, Seq1_30 mL/min_, and Seq2_30 mL/min_). Each arm was further divided into 2 dose groups: nominal (1×) and upper bound (2×), effectively doubling the study arms for a total of 6 groups. Groups 1 and 2 used Seq1 with 4 mL/min irrigation (Seq1_4 mL/min_) at a 1× dosage consistent with the manufacturer’s instructions for use, and 2× dose, respectively. Groups 3 and 4 used Seq2_30 mL/min_ at 1× and 2× doses, and groups 5 and 6 used Seq1 with 30 mL/min irrigation flow rate (Seq1_30__mL/min_) at 1× and 2× doses. Animals were treated with ablations at the right superior PV, right inferior PV, roof/posterior wall, left atrial appendage, right atrial lateral wall, and the mitral annulus (Figure 2). The left PVs were excluded owing to substantial anatomic differences between porcine and human left PVs that limit clinical translatability.^10^ To assess acute PVI, animals underwent bipolar voltage mapping procedures before and after ablation on day 0 using a diagnostic catheter (PentaRay Nav mapping catheter, Biosense Webster, Inc, part of Johnson and Johnson MedTech; Irvine, CA) and the CARTO 3 Navigation System (Biosense Webster, Inc, part of Johnson and Johnson MedTech; Irvine, CA). All animals were then survived for 28±2 days postablation to undergo follow-up bipolar voltage remapping procedures. Animals were euthanized after these assessments.

**Figure 2.**
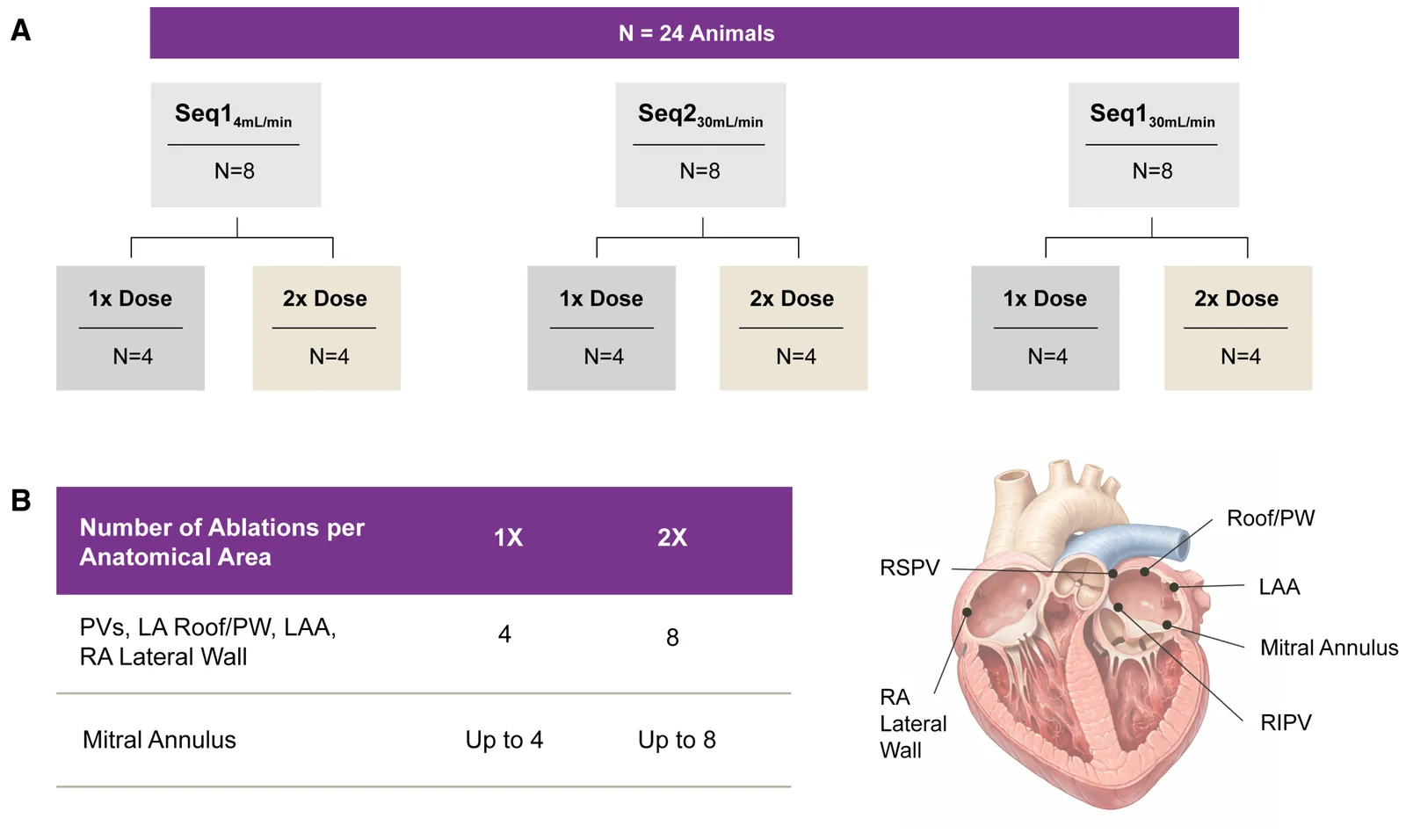
**Study design.** Schematic of (**A**) study design and (**B**) ablation strategy. LAA indicates left atrial appendage; LA Roof/PW, left atrial roof/posterior wall; PV, pulmonary vein; RSPV, right superior pulmonary vein; RIPV, right inferior pulmonary vein; and RA wall, right lateral wall. Image is courtesy of Biosense Webster, Inc, part of Johnson & Johnson MedTech. © All rights reserved.

### Safety End Points

Safety end points included monitoring phrenic nerve function during each ablation with postprocedure pacing and documented right phrenic nerve capture to confirm integrity.

Intraoperative imaging, including intracardiac echocardiography, was used to detect mural thrombus, pericardial effusion, and to measure PV diameter before ablation, with repeat assessment on day 28±2 to identify PV stenosis.

Continuous ECG monitoring looked for signs of acute ischemia consistent with coronary spasm and for ventricular fibrillation, including ventricular fibrillation associated with asynchronous pulse delivery.

Postprocedure safety assessments included microscopic inspection of catheter tips for blood thrombus coagulum and charring. Gross and histopathologic examinations of the heart and adjacent structures (ie, aorta, lungs, pulmonary artery, bronchi, coronary arteries, esophagus, phrenic nerves, and pericardium) were performed to identify collateral injury, intracardiac tissue damage, peripheral thromboemboli, and coronary artery injury. New neurological deficits and transient ischemic attacks were evaluated by clinical and neurological examination during the in-life phase.

Plasma and urine samples were collected and subjected to clinical pathology before ablation on day 0, immediately postablation on day 0, and on day 28±2. Clinical blood and urine chemistry tests were performed on all plasma and urine samples. Hemolysis was assessed by plasma-free hemoglobin (PFH). As defined by Shinohara et al,^11^ hemolysis was classified as mild, moderate, or severe based on PFH levels (30–49, 50–100, and >100 mg/dL, respectively).

Microbubble intensity during ablations was assessed by visual inspection of real-time intracardiac echocardiography and qualitatively scored by the operating physician as few, moderate, or shower as previously described in Kilicaslan et al.^12^

### Efficacy End Points

Acute and chronic electrical efficacy were defined as confirmation of bidirectional block (entrance and exit block) in the right and left atria, verified by a mapping catheter at the index procedure and at day 28±2, respectively. PV lesion circumference and contiguity were assessed grossly and microscopically by the study pathologist and recorded as the proportion of targeted PV ostial circumference exhibiting ablation effect and the presence or absence of gaps. Transmurality and contiguity of all lesions were further evaluated to assess lesion formation at the different target sites (ie, right superior PV, right inferior PV, roof/posterior wall, left atrial appendage, right atrial lateral wall, and mitral annulus). Transmurality scores were characterized by a 1 to 4 scale: 1≤25% of the wall affected; 2=25% to 50%; 3=50% to 75%; and 4=75% to 100%. Histological tissue response and treatment efficacy were evaluated grossly and microscopically by the study pathologist, documenting necrosis, fibrosis, inflammation, endothelial integrity, thrombus, and lesion maturation. Semiquantitative lesion scores were assigned from 0 (not present) to 4 (most severe). Scores from each PFA dose and pulse recipe cohort were combined to generate an overall mean rating.

### Statistical Analysis

Owing to the small sample size, exploratory nonparametric analyses were performed for selected end points. Changes from preablation to postablation for continuous variables (eg, PV ostial diameter reduction, PFH, and CK [creatine kinase]) were compared among treatment sequences separately within the 1×- and 2×-dose cohorts using the Kruskal-Wallis test. Changes from preablation to terminal measurements were also compared across all 6 study groups using the Kruskal-Wallis test. Prespecified Mann-Whitney *U* tests were used to compare outcomes between the combined 1×-dose groups and combined 2×-dose groups. Microbubble grades (few, moderate, or shower) were compared using the χ^2^ test. These statistical analyses were performed using MiniTab (Version 21.4.2), and a 2-sided *P* value of <0.05 was considered statistically significant. High-level binary end points, occurrence of adverse events, transmurality rate, and presence of char, as well as histology ratings were assessed using Fisher exact tests performed in R (Version 4.5.3; R Foundation for Statistical Computing, Vienna, Austria).

## Results

### Safety

Neither coronary vasospasm, mural thrombus, clinically relevant PV stenosis, phrenic nerve impairment, cardiac effusion, nor tamponade were noted for the duration of the study. No evidence of steam pop detected in any of the enrolled animals. In addition, phrenic nerve capture after pacing was successful in all animals, indicating no detectable loss of phrenic function. PV stenosis as assessed on intracardiac echocardiography demonstrated no clinically relevant acute or chronic reduction in PV diameter for any animals after ablation (Figure 3). No statistically significant differences in percent reduction in PV diameter were detected among the 6 groups (Kruskal-Wallis, *P*=0.943).

**Figure 3.**
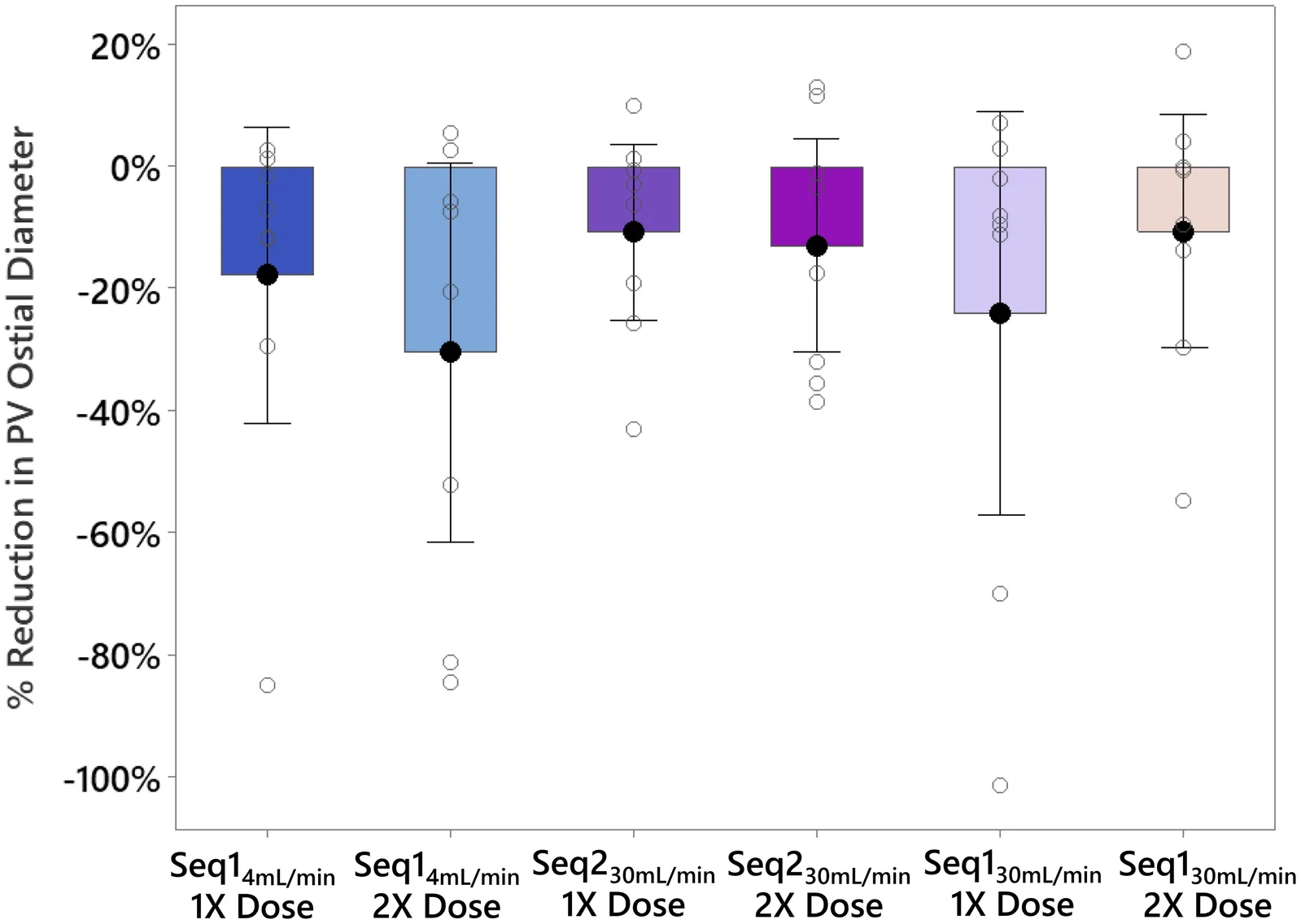
**Pulmonary vein (PV) diameter representation.** Bar graph of percent chronic (day 28±2) reduction in PV ostial diameter among all groups. Solid circles represent the mean percentage reduction in PV ostial diameter. Open circles represent individual data points. Interval bars represent 95% CIs. Percent reduction was defined as (acute−chronic)/acute×100; thus, negative values reflect an increase in diameter expected with normal growth rather than stenosis over the survival period.

### Gross Pathology

Within treatment sites, endocardial ablation across all groups after 28±2 days of recovery resulted in grossly evident, typically transmural ablation of cardiac tissue, with variably contiguous but overlapping ablation areas encompassing up to 80% of the left atrial endocardial surface. There was no gross evidence of treatment-related unintended tissue damage (ie, cardiac laceration or perforation) to the heart or significant collateral injury or peripheral organ effects (ie, esophageal injury, thromboemboli, and necrosis).

No collateral injury to surrounding anatomic structures including the aorta, lungs, esophagus, pericardium, or phrenic nerve was noted regardless of animal, treatment dose, PFA sequence, or treatment site location (Figure 4).

**Figure 4.**
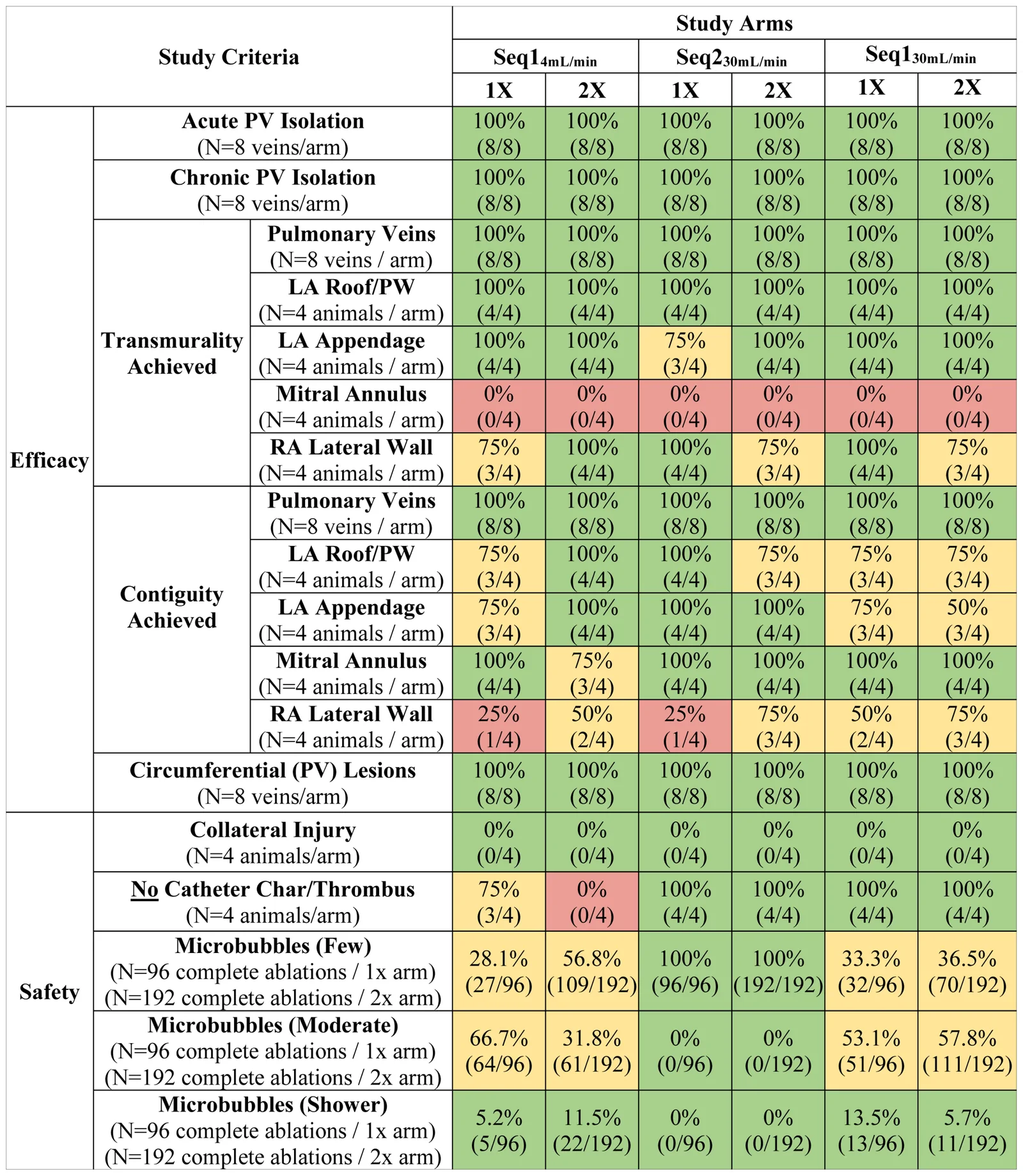
**Heat map showing summary of dose responses.** Percentages represent the within-group proportion meeting each study criterion. Green denotes favorable outcomes, yellow denotes intermediate outcomes, and red denotes less favorable outcomes. Collateral injury is defined as esophageal injury, phrenic nerve injury, and injury to upstream and downstream tissues. LA indicates left atrium; PV, pulmonary vein; PW, posterior wall; and RA, right atrium.

### Histopathology

Muscle fiber loss and lesion transmurality were extensive across all groups, with all PV lesions fully transmural, contiguous, and circumferential (Figure 5). Ablated cardiac tissue was replaced by fibrosis in amounts consistent with PFA lesions. Nerve degeneration remained minimal across all groups. Inflammation scores were significantly higher in the 2×-dose groups compared with the 1×-dose group as assessed by inflammation and eosinophil scores (*P*<0.001 and *P*<0.001); however, minimal inflammation and mineralization were observed overall (Figure 6). All groups had mild-to-moderate reactive tissue response, with the 2× dose groups having significantly higher scores of neointima, fibrosis, and neovascularization (Fisher exact test, *P*=0.03, *P*=0.01, and *P*=0.04; Figure 6). No statistically significant differences were detected among the three 1×-dose groups or three 2×-dose groups for transmurality, muscle fiber loss, fibrosis, neointima formation, neovascularization, or eosinophils (Fisher exact test, all *P*>0.05). Among the 2× dose groups, inflammation was significantly higher with Seq1_4 mL/min_ (Fisher exact test, *P*=0.02); however, no significant difference was observed among the 1×-dose groups (Fisher exact test, *P*=0.40).

**Figure 5.**
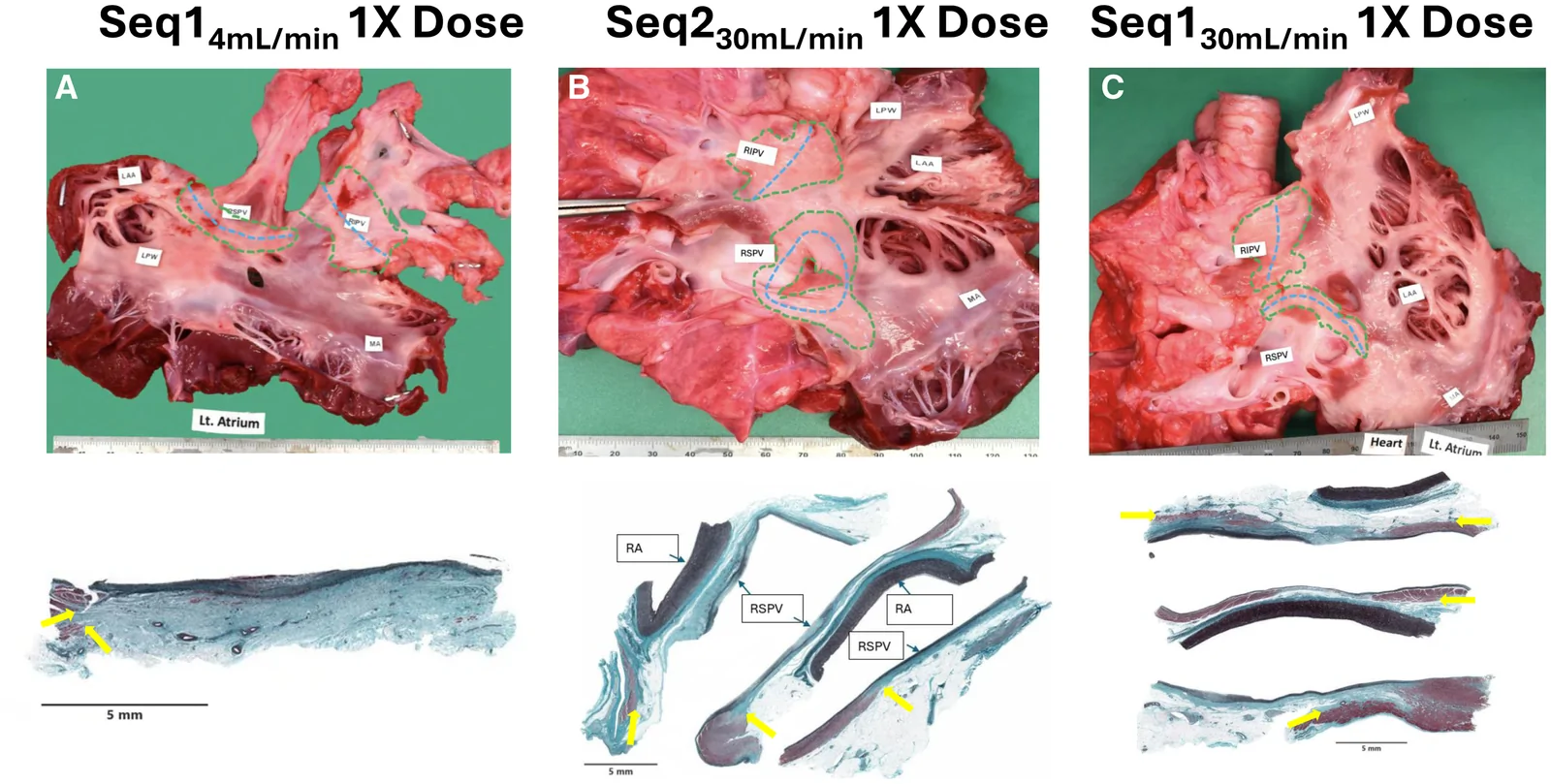
**Representative gross pathological and histopathologic lesion.** Images from (**A**) Seq1_4 mL/min_, (**B**) Seq2_30 mL/min_, and (**C**) Seq1_30 mL/min_. Gross pathology images show the left atrium. Green dotted lines highlight lesions, demonstrating circumferential/contiguous lesion bands in each pulmonary vein (PV). Blue dotted lines highlight the circumferential direction of the PV sleeves, some of which are cut as part of the macroscopic analysis. The lesions indicated by the green dotted line encompass the PV sleeves. In gross image **A**, the bright red color inside the lesion indicates acute hemorrhage due to the mapping catheter irritating the endocardium, not live tissue or gaps in the lesion band. Gomori’s Elastin Trichrome (GET) staining was used for histopathology images. Yellow arrows point to borders between ablated and nonablated myocardium. Blue color indicates fibrosis replacement of lesions. Red color indicates healthy myocardial cells. Contiguous (circumferential) and transmural lesions are shown in each image to represent 100% pulmonary vein isolation efficacy. LAA indicates left atrial appendage; LPW, left posterior wall; MA, mitral annulus; RA, right atrium; RIPV, right inferior pulmonary vein; and RSPV, right superior pulmonary vein. Image is courtesy of Biosense Webster, Inc, part of Johnson & Johnson MedTech. © All rights reserved.

**Figure 6.**
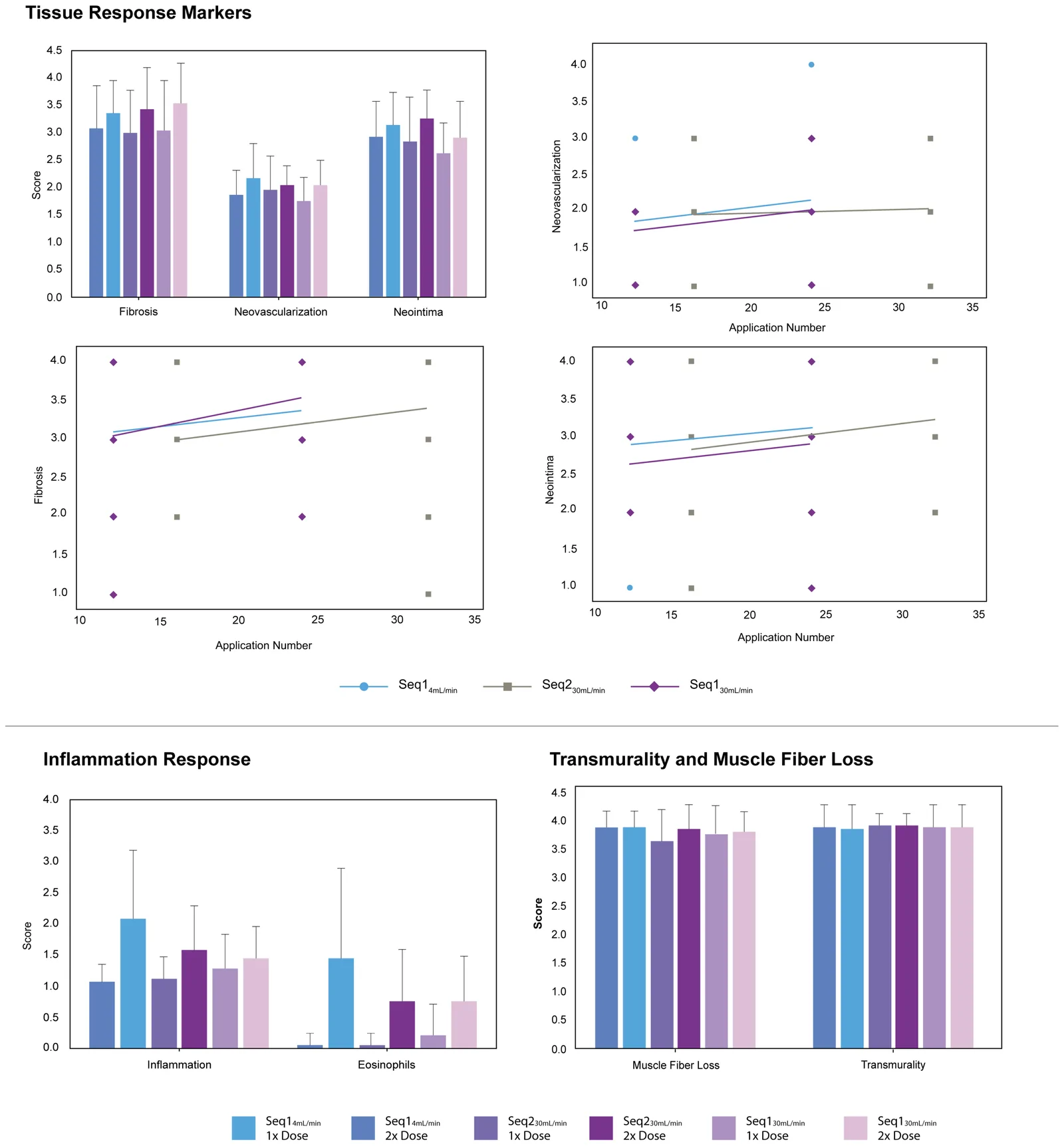
**Histology results.** Bar graphs comparing scores for (**A**) inflammation, (**B**) muscle fiber loss, and transmurality (**C**) reactive tissue response. Scoring: 0, not present; 1, minimal, present but slight feature; 2, mild, notable feature, not effacing or disrupting preexisting tissue elements, or limited to a small tissue area; 3, moderate, prominent or relatively widespread feature that does not disrupt tissue architecture, and is not overwhelming; and 4, marked/severe, overwhelming feature that is very widespread or complete, or that effaces or disrupts tissue architecture.

### Assessment of Hemolysis

Overall, across all Seq1 and Seq2 groups, there was an observed increase in LDH (lactate dehydrogenase), total bilirubin, indirect bilirubin, and PFH with decreases in haptoglobin and red cell mass when comparing the day 0 preablation and postablation time points. All markers generally returned to baseline levels by day 28±2 (Figure 7; Table).

**Table.**
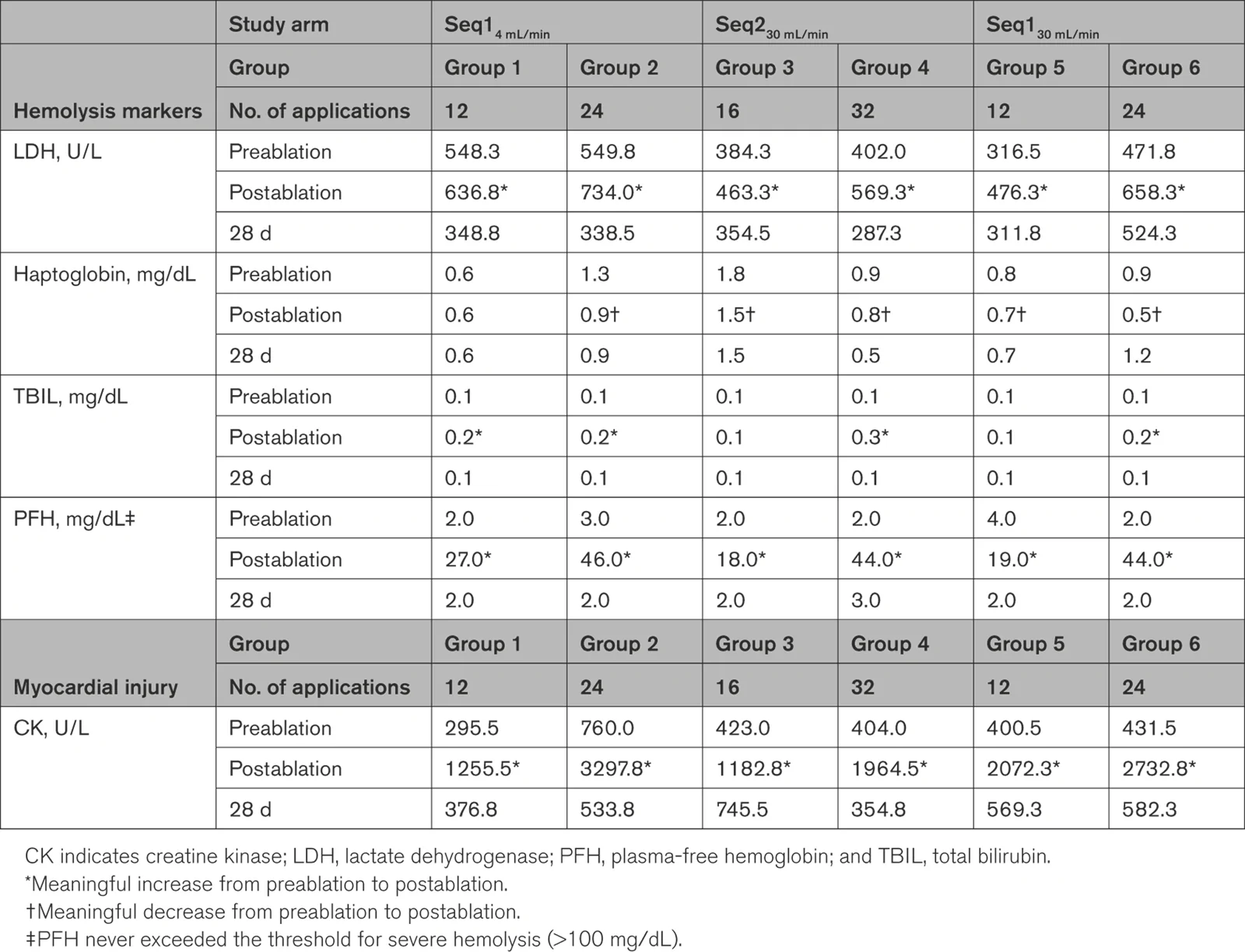
Hemolysis and Myocardial Injury Summary (Means)

**Figure 7.**
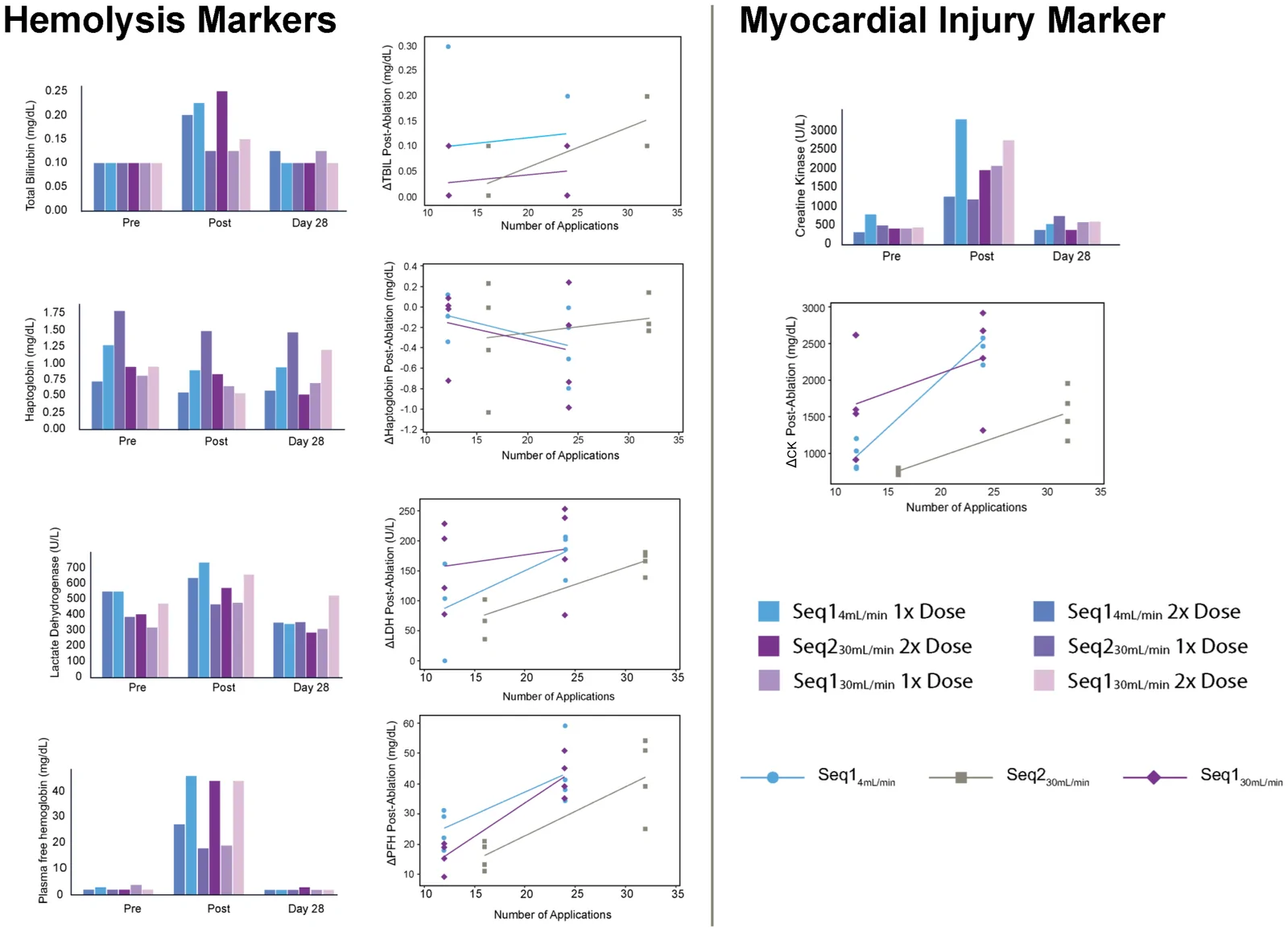
**Myocardial injury and hemolysis.** Dosage and temporal dynamics graphs in hemolysis and myocardial injury markers. LDH indicates lactate dehydrogenase; PFH, plasma-free hemoglobin; and TBIL, total bilirubin.

No significant differences in PFH were observed among treatment sequences (ie, Seq1_4 mL/min_, Seq2_30 mL/min_, and Seq1_30 mL/min_) within either the 1×- or 2×-dose cohorts (Kruskal-Wallis, *P*=0.124 and *P*=0.981, respectively). PFH levels were significantly higher in the 2×-dose groups than in the 1×-dose groups (Mann-Whitney *U* test, *P*<0.001). No severe hemolysis (PFH>100 mg/dL) was observed at any time point for any group.^11^ However, mild hemolysis (PFH, 30–49 mg/dL) was noted across all 2× dosing groups (Figure 7; Table). Specifically, a transient rise in PFH concentration to 46, 44, and 44 mg/dL on day 0 postablation for Seq1_4 mL/min_, Seq2_30 mL/min_, and Seq1_30 mL/min_, respectively, was observed. Recovery from procedure-related hemolysis, assessed by the change in PFH from baseline to terminal evaluation, did not differ significantly among the 6 study groups (Kruskal-Wallis, *P*=0.820; Table).

### Assessment of Myocardial Injury

Differences in acute CK elevation were observed among treatment sequences within both the 1×- and 2×-dose cohorts (Kruskal-Wallis, *P*=0.012 and *P*=0.077). CK levels were significantly higher in the 2× groups than in the 1× groups (Mann-Whitney *U* test, *P*=0.001). Recovery of CK toward baseline, assessed by the change from baseline to terminal evaluation, did not differ significantly among the 6 study groups (Kruskal-Wallis, *P*=0.230; Figure 7; Table).

### Microbubbles

In Seq2_30 mL/min_ (1× and 2× groups), 288 complete ablations were delivered, and every ablation (100%) received a few score. For Seq1_30 mL/min_, ablations were predominantly few or moderate, with 86.5% of the 1× group and 94.3% of the 2× group falling into that combined category. Similarly, in the Seq1_4 mL/min_ group, ablations were mostly few or moderate, with 94.8% of the 1× group and 88.6% of the 2× group in that combined category (Figure 4). A statistically significant difference was observed in microbubble intensity distribution among the 6 groups (χ^2^, *P*<0.001). The number of ablations did not seem to change the distributions of microbubbles.

### Charring

No catheter char or thrombus was noted in the Seq2_30 mL/min_ groups or the Seq1_30 mL/min_ groups; however, a significantly higher incidence was observed in the Seq1_4 mL/min_ group, with minimal catheter thrombus noted in 25% and 100% with the 1× and 2× dosages, respectively (Fisher exact test, *P*=0.003; Figure 4; Figure S1).

### Effectiveness

After the initial ablation procedure, diagnostic mapping confirmed complete elimination of PV potentials, indicating 100% acute effectiveness in the left atrium for all animals (Figure 8). Both Seq1 and Seq2 workflows similarly resulted in 100% chronic (28±2 days) PV isolation and consistently produced 100% transmural, circumferential lesions as confirmed by histopathology. At the left atrial roof, 100% transmurality was observed for all subjects. At the left atrial appendage and right atrial lateral wall, transmurality was achieved in at least 75% of samples across all groups and absent at the mitral isthmus (Figure 4).

**Figure 8.**
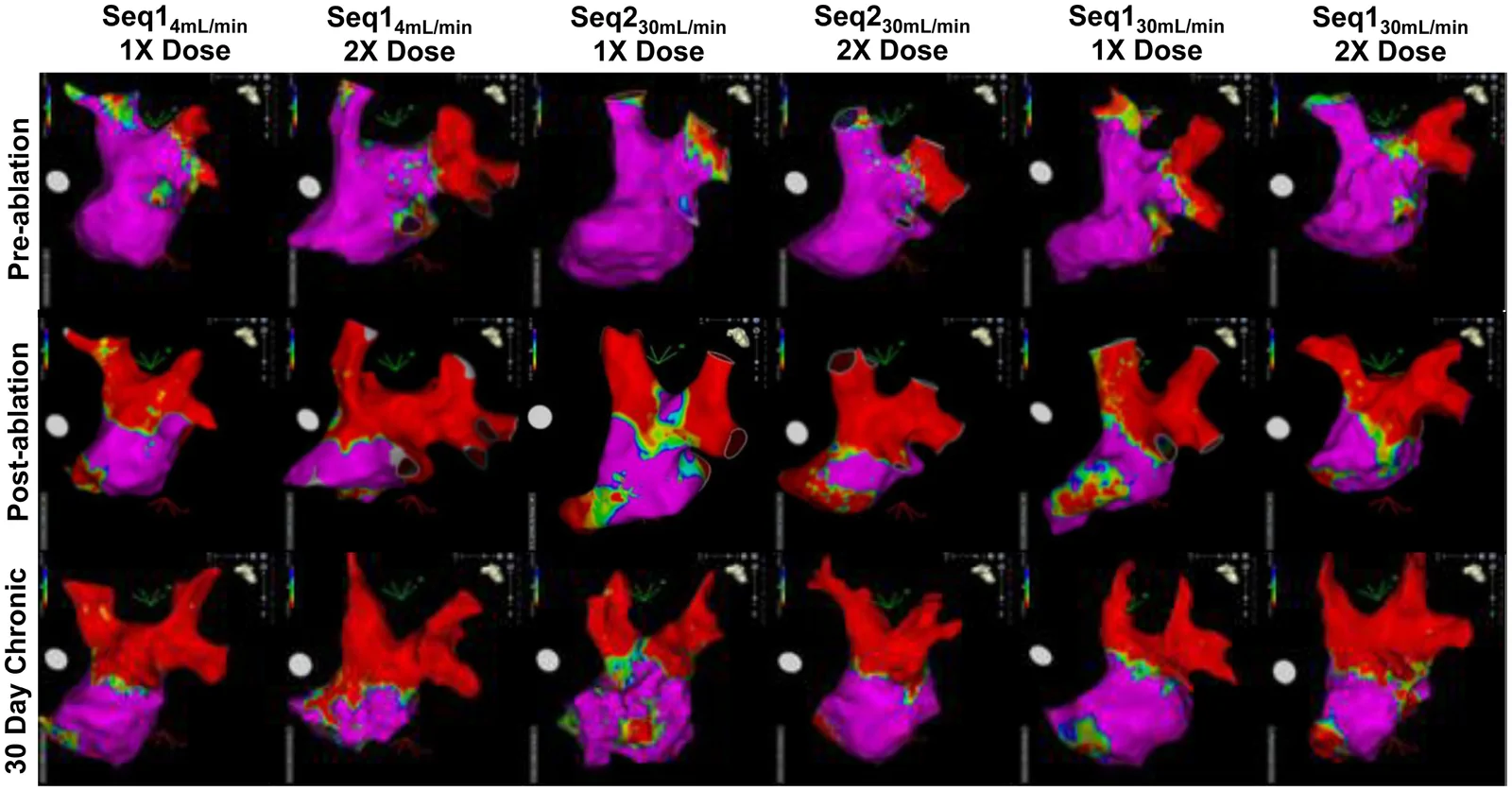
**Voltage maps by study arm.** Voltage maps and electrogram readouts preablation, postablation (acute), and after day 28±2 (chronic). Voltage is represented on a color scale from purple (high voltage, healthy tissue) to red (low voltage, scar tissue). Image is courtesy of Biosense Webster, Inc, part of Johnson & Johnson MedTech. © All rights reserved.

## Discussion

This Good Laboratory Practice study sought to gather preclinical evidence that the second-generation VLCC pulse waveform, Seq2_30 mL/min_, maintains efficacy and enhanced safety and performance compared with the predecessor, Seq1 (Graphic Abstract).^13–15^

After PFA with Seq1 and Seq2, histopathology assessment revealed extensive muscle fiber loss and lesion transmurality across all groups, with all PV lesions fully contiguous and circumferential. Ablated cardiac tissue was replaced by fibrosis in amounts consistent with PFA lesions, and nerve degeneration remained minimal across all groups. These findings suggest that Seq2_30 mL/min_ preserves the core electroporative mechanisms necessary for durable lesion formation as evidenced by 100% acute and chronic PV isolation, consistent with lesion outcomes previously reported for the validated PFA VLCC waveform.^15,16^ Interestingly, efficacy results did not vary depending on 1× or 2× the therapeutic dosing, suggesting that nominal dosing with either sequence produces equivalent lesion formation to the 2× dose in the porcine model. Hence, there was no additional therapeutic benefit observed from applying a 2× dose within the context of this model.

In this study, PVI achieved a 100% transmurality rate, consistent with the relatively uniform geometry and favorable electrode–tissue coupling in these regions.^17^ In contrast, transmurality was intermediate at the left atrial appendage and right atrial lateral wall and was not achieved at the mitral annulus. These findings likely reflect regional variation in atrial wall thickness and architecture that modulate effective field penetration.^15,17,18^ Although left atrial appendage thickness is variable, it is generally greater and structurally more complex than the posterior wall and PVs, where target tissue thickness is typically <2 mm.^17^ By comparison, the left atrial ridge and roof are commonly ≈5 to 7 mm thick, and the mitral annulus spans ≈2.5 to 8 mm, underscoring substantial spatial heterogeneity.^17^ In the MA, tissue thickness coupled with the presence of epicardial connections may further limit effective field penetration to deeper tissue layers. Collectively, greater thickness and structural complexity likely contributed to the lower and more heterogeneous transmurality observed in the other target regions relative to the thinner, more uniform PV regions. These preclinical results at the MA are reminiscent of clinical results that have been reported for the pentaspline catheter, which exhibited a lack of long-term durability.^19^

Each PFA application delivers high-voltage pulses that can electroporate red blood cell membranes; repeated exposures can cumulatively increase red blood cell injury and lysis risk.^20^ In this study, there were no incidences of moderate or severe hemolysis, even under conditions of elevated cumulative pulse delivery. The observation of mild hemolysis in the 2×-dose groups, compared with minimal hemolysis in the 1×-dose group, further corroborates a dose-dependent hemolytic effect (ie, an increase in the number of PFA applications leads to an increased degree of hemolysis) as previously observed in both clinical and preclinical settings.^21–23^ Notably, in independently reported patient studies, even when utilizing the lower irrigation rate (4 mL/min), the VLCC demonstrated lower per-application hemolysis than a widely used PFA system with a pentaspline electrode configuration, highlighting that waveform and catheter design (eg, smaller active electrode areas and optimized tissue contact) can mitigate hemolysis even when irrigation is limited.^9,24,25^ Although human studies suggest that physical displacement of red blood cells (eg, via higher irrigation) may attenuate hemolytic effects, in this study, minimal hemolysis was observed with both low (4 mL/min) and high (30 mL/min) irrigation, with no observable difference among any of the 1×-dosed groups.^9,24^ This lack of concordance with human studies may be explained by differences between humans and the porcine model, and future clinical studies are needed to confirm whether the higher irrigation rate and waveform may indeed attenuate hemolysis in humans. Overall, these findings indicate that the VLCC has a favorable hemolysis safety profile across both waveforms and irrigation rates, suggesting that Seq2_30 mL/min_ is well tolerated under both 1× and 2× the therapeutic recommendation.

The thromboembolic profile of Seq2_30 mL/min_ was assessed in this study. Earlier benchtop testing demonstrated the thermal advantage of using a higher irrigation rate with the VLCC, which subsequently led the manufacturer to update the Seq1 user instructions to recommend 30 mL/min to reduce the risk of thromboembolic events.^4^ Zito et al^8^ further corroborated these findings in their investigation comparing temperatures across Seq1_30 mL/min_, Seq2_30 mL/min_, and Seq1_4 mL/min_, reporting lower temperatures for both the Seq1_30 mL/min_ and Seq2_30 mL/min_ waveform. Accordingly, the absence of catheter thrombus observed in the present study with the Seq1_30 mL/min_ and Seq2_30 mL/min_ groups may be partly explained by these lower temperatures, highlighting a potential mechanistic advantage of combining the optimized Seq2_30 mL/min_ pulse waveform with the higher irrigation flow.^4,8^

Visual intraoperative microbubble assessment demonstrated that the majority of all groups had few to moderate microbubbles. Notably, all microbubble observations in the 1× and 2× Seq2 groups were classified as few, with no instances of moderate or a shower of microbubbles recorded. This is consistent with prior work showing that microbubble formation during PFA is waveform dependent. Waveform parameters (eg, pulse duration and repetition pattern) alter electrode charge delivery and local gas-evolution dynamics, a relationship observed in both in vitro and intracardiac PFA studies.^17,26,27^ Seq2_30 mL/min_ combines shorter pulse packets and application time. Mahnič-Kalamiza et al^27^ showed that short pulse phase lengths decrease faradaic currents and bubble formation; as such, the reduced pulse length and exposure time in Seq2_30 mL/min_ may further restrict the opportunity for microbubble growth. In addition, Seq2_30 mL/min_-associated lower electrode/surface temperature, as demonstrated by Zito et al,^8^ suggests a lower likelihood of microbubbles that may generate as thermal byproducts.^27^ Accordingly, the slightly reduced microbubble burden observed in the Seq2_30 mL/min_ groups may reflect both its limited thermal effects and inherent differences in waveform design, including shorter pulse packets and application duration compared with Seq1.

### Study limitations

This study is limited by the inherent constraints of preclinical animal models, including unknown translation to humans, as well as potential variability in PV anatomy and branching. The relatively small sample size reduced our ability to identify rare adverse events. Although nonparametric analyses were performed due to the limited sample size inherent to an in vivo, large animal study, these findings should be interpreted cautiously. Microbubble assessment was qualitative, allowing descriptive comparisons but not the precision of quantitative ultrasonic bubble-counting techniques; magnetic resonance imaging was also not performed to quantify cerebral microembolization, which limits evaluation of embolic risk. Because coronary angiography was not performed systematically, asymptomatic coronary spasm cannot be entirely excluded. However, the animals were continuously monitored with electrocardiography, which would likely have detected clinically significant ischemic changes, including ST-segment elevations.

### Conclusions

In this preclinical study, durable PVI was achieved across all groups, including the newly developed Seq2_30 mL/min_ waveform, at both 1× and 2× therapeutic dosing conditions. Mild hemolysis was observed in all groups. In addition, the arms with higher irrigation (Seq2 and Seq1_30 mL/min_) achieved zero catheter charring. Lastly, Seq2 was associated with few microbubbles at both 1× and 2× dosing. Taken together, these data support safe and effective integration of shortened ablation delivery time from 20.6 to 3.8 seconds. Additional clinical testing is required for further validation.

## ARTICLE INFORMATION

### Acknowledgments

The authors thank Amanda McCormac for scientific writing.

### Disclosures

Dr Di Biase is a consultant for Biosense Webster, Inc, part of Johnson and Johnson MedTech, Boston Scientific, Stereotaxis, and Abbott and has received speaker honoraria from Medtronic, Zoll, and Biotronik. Dr Hsu reports receiving honoraria from Medtronic, Abbott, Boston Scientific, Biotronik, Janssen Pharmaceuticals, Bristol Myers Squibb, Pfizer, Sanofi, Altathera. Pharmaceuticals, Milestone Pharmaceuticals, Zoll Medical, iRhythm, Edwards Lifesciences, vizai, Atraverse Medical, Arga Medtech, and Biosense Webster, Inc, part of Johnson and Johnson MedTech, and has equity interest in Vektor Medical. Dr Bhardwaj has received honoraria and consulting fees from Biosense Webster, Inc, part of Johnson and Johnson MedTech. Dr Newton is a consultant for Atricure. Dr Maddox is a consultant for Biosense Webster, Inc, part of Johnson and Johnson MedTech, Medtronic, Abbott, and Boston Scientific. Dr Sauer has received grants from Biosense Webster, Inc, part of Johnson and Johnson MedTech, and Medtronic; and consulting fees from Boston Scientific, Biosense Webster, Inc, part of Johnson and Johnson MedTech, and Abbott. Dr Romero is a paid consultant for Biosense Webster, Inc, part of Johnson and Johnson MedTech, Boston Scientific, AtriCure, Abbott, and Sanofi. Drs Gomez, Chen, E. Byun, and C. Beeckler are employees of Biosense Webster, Inc, part of Johnson and Johnson MedTech. The other authors report no conflicts.

### Supplemental Material

Figure S1

## Supplementary Material
