# Supplementary material for: Effect of Pulsed Field Timing Sequence and Electrode Cooling on Ablation Safety and Efficacy Surrogates: A Preclinical Study
Source: Circ Arrhythm Electrophysiol. 2026 Sep 23;19(10):e015042. doi: 10.1161/CIRCEP.126.015042 (PMC13686642; doi:10.1161/CIRCEP.126.015042)
Supplement: Supplementary file 1 [file hae-19-e015042-s001.pdf]

Supplemental Material

Supplemental Figures with Figure Legends

Seq1$_{4mL/min}$ 1X Dose Thrombus Score 1

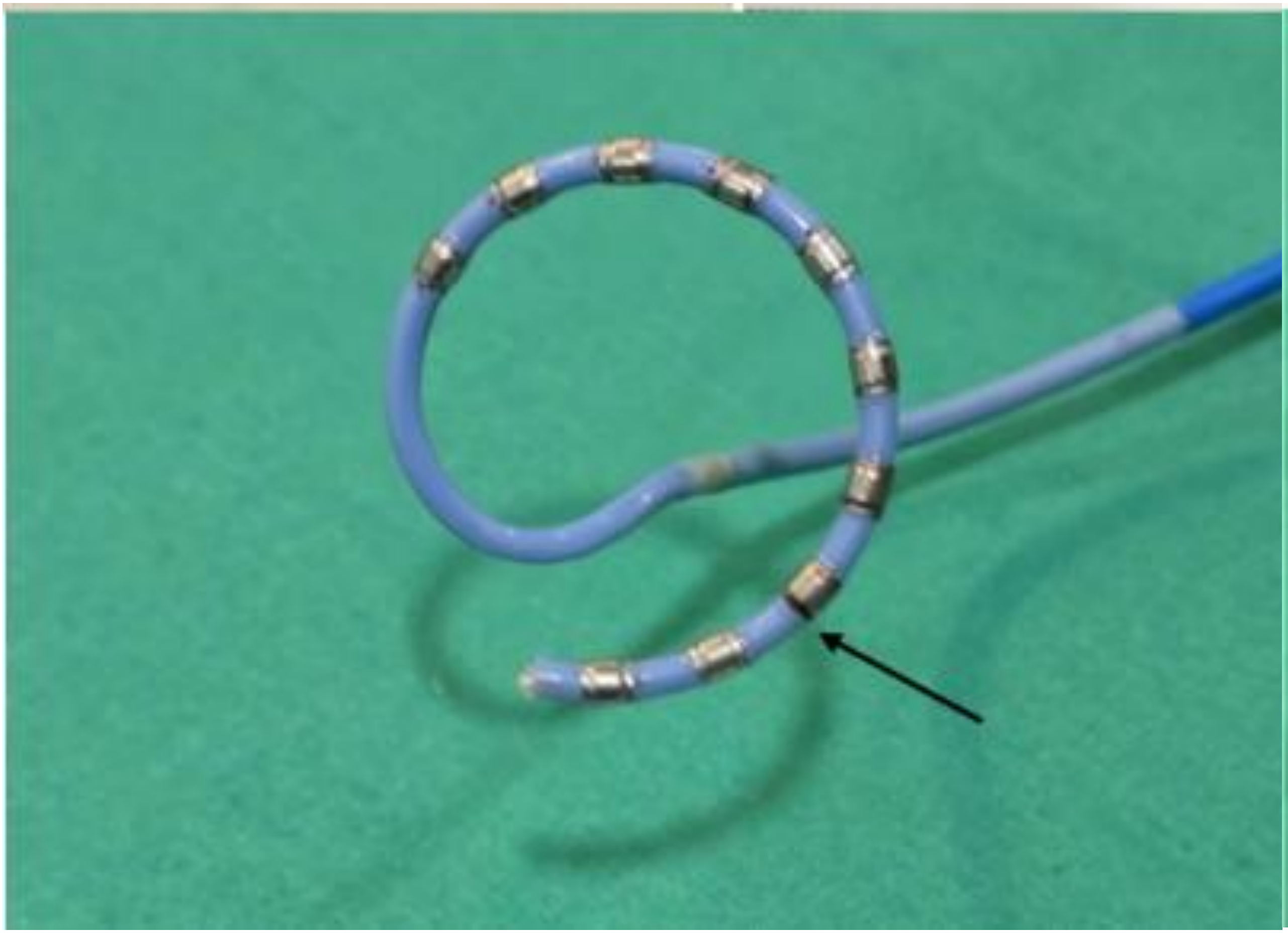

Seq1$_{4mL/min}$ 2X Dose Thrombus Score 1

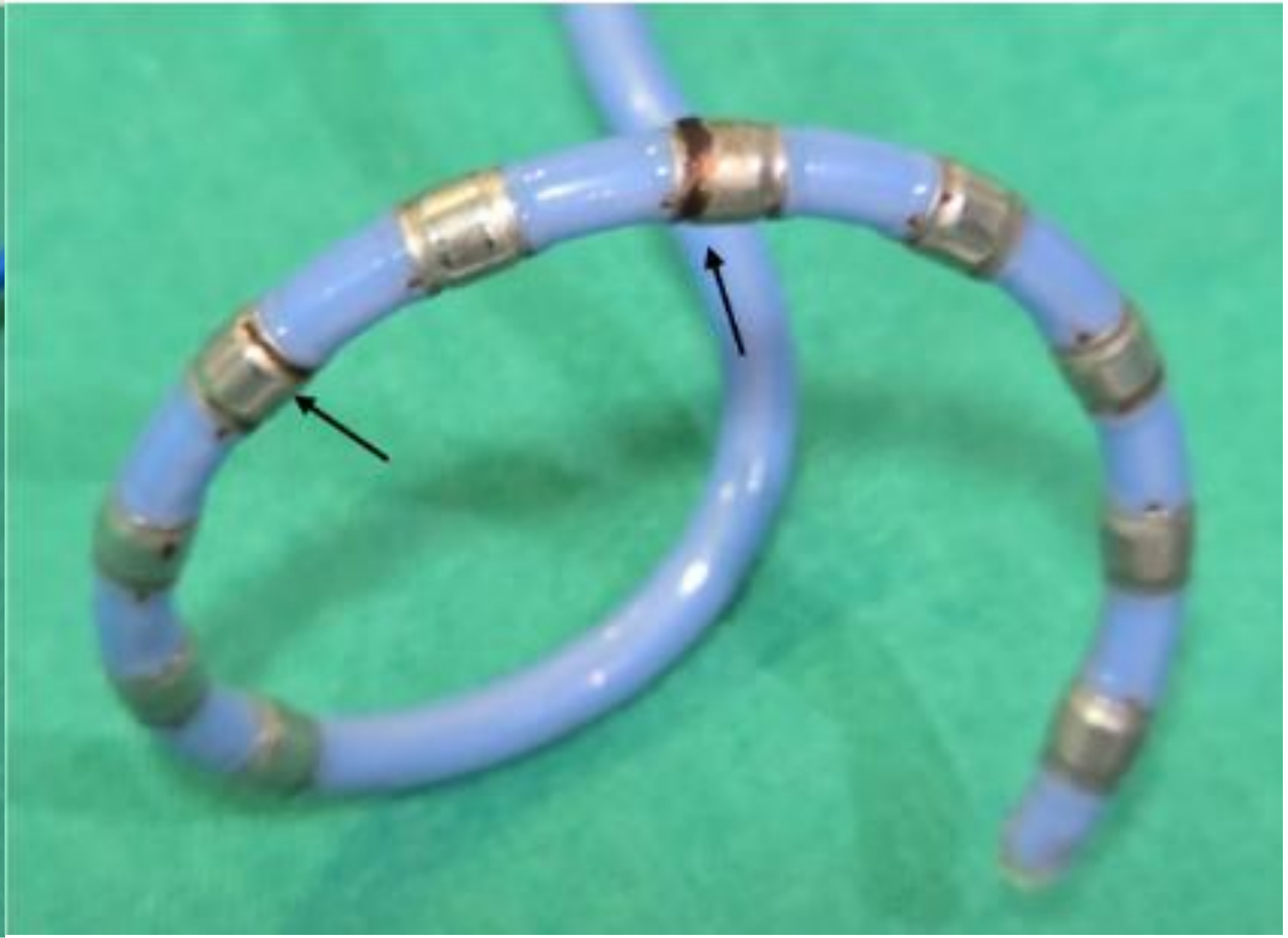

Supplemental Figure 1: Representative images of catheter thrombus with $Seq1_{4mL/min}$ at 1X dose and 2X dose. Arrows indicate where thrombus is visualized.
